# Epstein-Barr Virus Mediated Signaling in Nasopharyngeal Carcinoma Carcinogenesis

**DOI:** 10.3390/cancers12092441

**Published:** 2020-08-28

**Authors:** Timmy Richardo, Pongphol Prattapong, Chawalit Ngernsombat, Nurulfitri Wisetyaningsih, Hisashi Iizasa, Hironori Yoshiyama, Tavan Janvilisri

**Affiliations:** 1 Institute of Virology, Hannover Medical School, 30625 Hannover, Germany; Richardo.timmy@mh-hannover.de; 2 Department of Biomedicine, Indonesia International Institute for Life Science (i3L), Jakarta 13210, Indonesia; n.wisetyaningsih@i3l.ac.id; 3 Department of Microbiology, Shimane University, Izumo 693-8501, Japan; iizasah@med.shimane-u.ac.jp (H.I.); Yosiyama@med.shimane-u.ac.jp (H.Y.); 4 Graduate Program in Molecular Medicine, Faculty of Science, Mahidol University, Bangkok 10400, Thailand; pongphol.pra@student.mahidol.ac.th (P.P.); chawalit.ngr@student.mahidol.ac.th (C.N.); 5 Department of Biochemistry, Faculty of Science, Mahidol University, Bangkok 10400, Thailand

**Keywords:** nasopharyngeal carcinoma, EBV, oncogenesis, infection, signaling pathway

## Abstract

**Simple Summary:**

Epstein-Barr virus (EBV) infection is known to contribute in nasopharyngeal carcinoma (NPC) carcinogenesis. The oncogenic roles of the EBV proteins and non-coding RNAs in NPC are becoming evident with the aid of current advances in genome-wide and in-depth molecular analyses. This current work provides a comprehensive overview, which covers recent understandings of the pathogenic role of EBV infection in NPC. Perspectives on molecular mechanisms, which are involved in the pathogenesis of NPC, focusing on the connection between EBV and NPC cells and the corresponding signaling pathways are highlighted. Cancer hallmarks associated with EBV in NPC development are also discussed herein.

**Abstract:**

Nasopharyngeal carcinoma (NPC) is one of the most common tumors occurring in China and Southeast Asia. Etiology of NPC seems to be complex and involves many determinants, one of which is Epstein-Barr virus (EBV) infection. Although evidence demonstrates that EBV infection plays a key role in NPC carcinogenesis, the exact relationship between EBV and dysregulation of signaling pathways in NPC needs to be clarified. This review focuses on the interplay between EBV and NPC cells and the corresponding signaling pathways, which are modulated by EBV oncoproteins and non-coding RNAs. These altered signaling pathways could be critical for the initiation and progression of NPC.

## 1. Introduction

Nasopharyngeal carcinoma (NPC) is a malignant cancer, which is located behind the nose at the very upper part of the throat. As its symptoms are usually silent and painless, the patients mostly are diagnosed in the advanced stages of the disease, leading to poor prognosis [1]. Not only do inherited genetic mutations pose one of the important causes of the cancer, the Epstein–Barr virus (EBV) also plays a key role in the onset of disease and progression of NPC [1,2,3]. EBV-associated carcinogenesis is a multi-step process, involving the accumulation of genetic alterations in the cells, leading to genetic instability and dysregulation of signaling pathways, which subsequently transforms the cells allowing EBV genome maintenance and leading to the acquisition of cancerous phenotypes [3]. The dysregulation of signaling pathways in NPC has been extensively studied [4,5]. Members of such pathways can be useful for the early detection, prognosis, and targeted therapy. This review article will focus on the contribution of EBV in NPC carcinogenesis and NPC progression with the emphasis on the role of EBV oncoproteins and non-coding RNAs on altering signaling pathways in NPC cells.

## 2. Epidemiology of NPC

**Figure 1:**
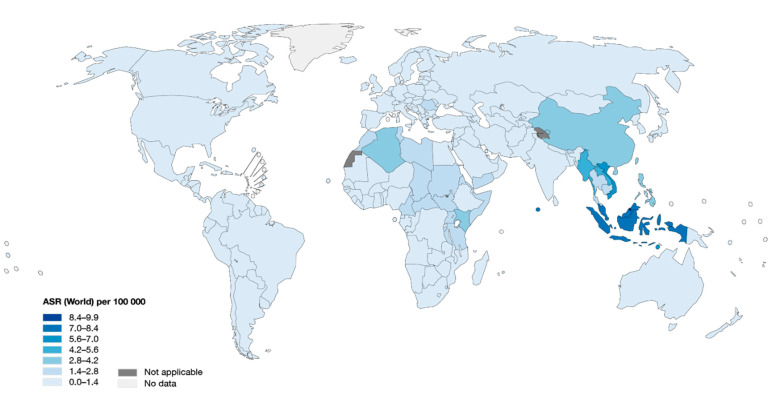
Global distribution of nasopharyngeal carcinoma. Nasopharyngeal carcinoma (NPC) incidence rate is represented as 100,000 person/years for all sex at all ages (0–85 years old). The map is obtained from International Agency for Research on Cancer (IARC) web-based cancer database, Global Cancer Observatory (GCO), and modified based on Globocan, 2018 [6].

In 2018, the global estimate of cancer incidence worldwide has been reported to be approximately 18.1 million cases, with 129,079 of those cases being NPC estimating an age-standardized rate (ASR) of 1.5 per 100,000 people [6]. In the same year, a total of 72,987 mortality of NPC has been reported, accounting for ~0.8% of the total cancer deaths. Comparing incidence to other types, NPC can be considered an uncommon cancer, and its distribution worldwide is very imbalanced (Figure 1). NPC is a rare cancer in the United States (ASR 0.45) and most European countries (ASR 0.44); however, it is much more common in Asia, especially in Southeastern (ASR 5.0) and Eastern Asian countries (ASR 2.7) where 27% and 50% of the total NPC cases have been reported in the two areas, respectively [7].

Considering the uneven distribution of NPC globally, risks related to ethnicities have been suggested to contribute as a prominent risk factor. Among others, Chinese descendants are usually at higher risk, with a considerably higher incidence compared to other ethnicities in the same area [2,8,9,10]. Large-scale case-control studies have revealed an increased risk in NPC development among the Chinese population, with an increased familial risk observed among relatives and a younger age of onset in families with higher affected cases [11,12,13,14]. Furthermore, consumption of traditional diets high in nitrosamines such as salted fish and preserved vegetables is a causative factor in NPC as well [15,16,17]. Inflammatory inducing diets are also noteworthy in increasing the risk of NPC [18]. In contrast, consumption of non-preserved vegetables and fruit is inversely associated to NPC and can be considered protective in effect [15,19,20,21]. On the other hand, lifestyle habits such as smoking has also been associated with NPC [17,22,23] in addition to a higher mortality in patients [24]. Nonetheless, familial risks of NPC seems to be retained regardless of the ancestry, with similar observation found in non-Chinese populations in the United States, Europe, and Australia [25], highlighting further the role of genetic determinants underlying the development of NPC.

Looking into the host genetics, human leukocytes antigen (HLA) containing the major histocompatibility complex (MHC) region on chromosome 6p21 is recognized as the major risk loci for NPC [26]. However, single nucleotide polymorphisms (SNPs) identified through whole-genome sequencing (WGS) in DNA extracted from EBV-positive NPC tissues might reflect mutations that occur during tumorigenesis and may not be the congenital mutations of the patients. Li et al. have shown that mutations in the MHC expression inducer NLRC5, MHC class I rearrangements, and point mutations accumulated during the growth of tumor cells [27].

Not only are mutations in genes involved in immune escape of tumor cells, mutations that strongly activate the nuclear factor kappa B (NF-κB) signal in tumor cells were also found [27]. TNFRSF19, CDKN2A/2B, MDS1-EVI1, and CLPTM1L/TERT were other susceptible genes [28,29]. The whole-exome sequencing (WES) data demonstrated that several rare genes might contribute to the development of NPC such as genes involved in Notch signaling (NOTCH1, DLL3), EBV entry into epithelial cells (ITGB6), EBV modulation (BCL2L12, NEED4L), magnesium transport (NIPAL1), DNA repair (PRKDC, MLH1), and cAMP signaling (RAPGEF3) [30].

Despite the high prevalence of NPC in Asian countries, the latest cohort study showed that NPC incidence has gradually declined [10,31]. A continuous declining pattern of NPC incidence over the last few decades may be related to the changes in lifestyle and advancement in healthcare facilities [32,33].

## 3. EBV Strains

EBV can be classified into type 1 and type 2 based on the differences in the EBV nuclear antigen 2 (EBNA2) gene sequence. The type 1 EBV (e.g., B95-8, GP202, and Akata) exhibits worldwide distribution, while type 2 EBV (e.g., AG876) is prevalent in Africa [34]. Recently, the genomes of EBV isolates collected from various locations are fully sequenced to investigate geographic variation and its association with diseases [34,35], as shown in Table 1. The EBV strain M81 is shown to infect epithelial cells more efficiently than EBV strains isolated from B lymphoma [36], suggesting that the cell tropism of a viral strain may correlate with the type of tumors.

The SNPs can be found in various EBV genes and transcription regulatory regions. Accordingly, latent membrane protein 1 (LMP1) genes are classified into six groups: China 1, China 2, Alaskan, Mediterranean, North Carolina, and B95-8 [37,38,39]. China 1 LMP1 strongly activates NF-κB signals compared to B95-8 LMP1 [39]. It was also reported that mutations in the BALF2 gene found in 83% of EBVs in South China were strongly correlated with NPC cases [40]. Further experiments using recombinant viruses are expected to confirm whether these SNPs are truly associated with NPC formation.

An SNP in the *Bam*H I A rightward transcript (BART) promoter region has been shown to induce *BART* promoter activity and high expression of BART miRNA is expected [41]. The SNP is strongly associated with NPC cases [41]. An SNP is also found in an EBV-encoded small non-coding RNA (EBER) gene from North Chinese NPC patients and the variation is different from EBV strains isolated from gastric carcinoma [42]. The V3 polymorphism in the Zp promoter activates EBV lytic infection in NPC [43].

## 4. EBV and NPC Carcinogenesis

Although EBV infection is pervasive, the interplay of EBV and other NPC risk factors is also evident as an increased risk is observed for EBV-seropositive with consumption of salted fish and other NPC environmental causes among high-risk groups [2,16,50].

### 4.1. EBV Life Cycle in Epithelial Cells

EBV is a ubiquitous gamma herpes virus, which infects greater than 90% of the world’s population [31,51]. EBV has been known as a causative agent for diseases such as Burkitt’s lymphoma, gastric carcinoma (GC), and NPC [52,53,54]. Primary EBV infection in epithelial cells begins when the virus crosses epithelial cells of the nasopharynx, infecting the naïve B cells in Waldeyer’s tonsillar ring [55]. It is believed that pharyngeal epithelial cells act as a reservoir, in which when the lytic reactivation is induced, the EBV genomes are amplified and viral particles are released into saliva for transmission.

**Figure 2:**
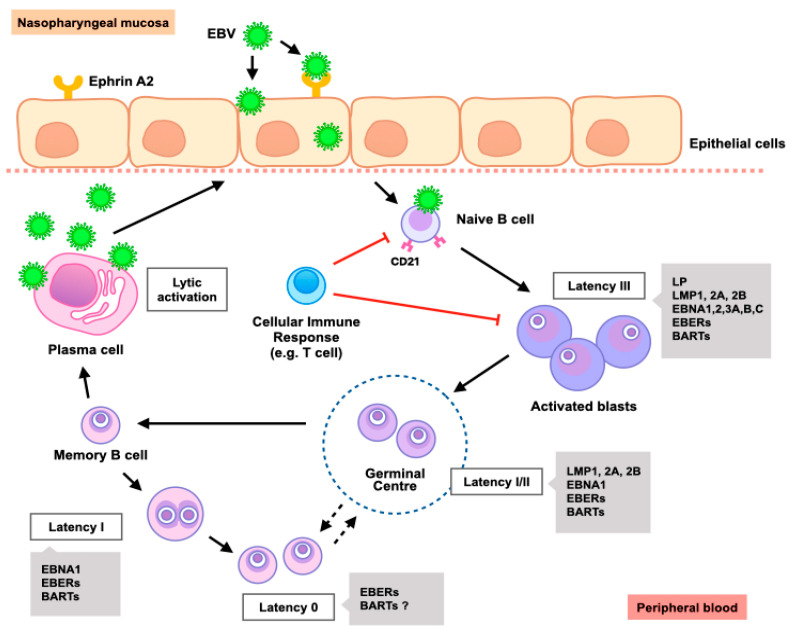
EBV life cycle. Primary EBV infection initiates in nasopharyngeal mucosa. EBV utilizes distinct glycoproteins to infect epithelial cells through Ephrin A2 and naïve B cells via the CD21 receptor. Viral entry causes the transport of the EBV genome into the B-cell nucleus, where viral replication process begins. EBV gene products activate the B-cell growth program (Latency III), resulting in the proliferation of blasting B cells. Normally, these blasting B cells are destroyed by cellular immune responses such as cytotoxic T lymphocytes. Once in the circulation, previously activated memory B cells may continue to undergo lytic replication or, if EBV shuts down most of its protein-encoding genes, it enters latency state. At a later time, as cells recirculate between the nasopharynx mucosa and peripheral compartments, memory B cells may be activated, resulting in viral reactivation and shedding. Genes expressed during each EBV latency period are indicated in the grey boxes.

The route of entry for EBV in epithelial cells is complicated. There are several hypotheses on how EBV enters epithelial cells, which have been reviewed elsewhere [56]. In normal pharyngeal epithelium, CD21 is weakly expressed or nearly absent [57]. It has been demonstrated that dysplastic pharyngeal epithelial cells expressed *CR2* gene (CD21) transcripts [58]. It is yet to be determined whether CD21 plays a role in EBV entry to epithelial cells. However, recent findings revealed that novel receptors mediated EBV entry into nasopharyngeal epithelial (NPE) cells such as neuropilin1 (NRP1) and ephrin A2. NRP1 interacts with EBV glycoprotein gB to facilitate viral internalization and membrane fusion [59,60], while ephrin A2 has been shown to induce the EBV infection with the cell-free virus in immortalized NPE cells [61,62]. The life cycle of EBV in nasopharyngeal cells is shown in Figure 2.

In order to understand how EBV induces tumors in epithelial cells, it is desirable to use non-transformed cells as an oncogenic model. Lymphoblastoid cell lines (LCL) represent a good tool to study the formation of EBV-associated lymphomas. However, EBV infection to primary epithelial cells induces lytic infection [63,64], therefore primary epithelial cells are not suitable for oncogenic study. Instead, cell lines originated from cancers are used, because these cell lines often show latent infection of EBV. It is well known that EBV-positive NPC tumor cells lose EBV episomes and become EBV-negative during in vitro culture [65]. EBV-positive NPC tumor cells can be isolated using the xenograft method of inoculating mice with NPC patient tissue. It is known that in vitro culture of NPC tumor cells shows lytic EBV infection, thus EBV-infected cells are killed and the remaining cells are all negative for EBV [66,67]. However, when the same cells were cultured with the addition of ROCK inhibitors, lytic infection was suppressed and EBV-positive cell lines could be established [66,67].

### 4.2. EBV Latency in NPC

EBV episomes replicate through the rolling circle method to generate multiple copies of the linearized EBV genome packaged into an infectious virus [68]. Upon infection, this linearized genome is circularized into episomes and heavily methylated to drive EBV into latency by limiting viral gene expression to a few coding and non-coding genes only. Homogenous length of terminal repeat is identified in NPC and pre-invasive lesions, indicating that EBV infection begins with clonal expansion of a single EBV-infected cell during the early stage of NPC development [69,70]. EBV persistently infects NPC cells as the latency II pattern that expresses episomal anchoring protein EBNA1, EBERs, BARTs, *Bam*H I-A rightward frame-1 (BARF1), LMP1, and LMP2A. In addition, BARTs are expressed abundantly in NPC, suggesting their essential role in malignancies [71].

EBNA1 regulates cellular gene expression through binding of the viral DNA to cellular promoters [72,73]. It activates the viral promoter Cp and Wp and inhibits Qp promoters [74], which are important for the viral fate determination. EBNA1 is also essential for viral genome maintenance, chromosomal destabilization, and immune evasion.

LMP2A together with LMP2B promote motility, epithelial cell migration, and suppress differentiation [75]. In NPC, LMP2A enhances the epithelial-mesenchymal transition (EMT) via the metastatic tumor antigen 1 (MTA1) and mammalian target of rapamycin signaling [76]. BARF1 may induce carcinogenesis by immortalizing and transforming epithelial cells, thereby enabling immune evasion and cell survival [77]. Interestingly, BARF1 is exclusively expressed in EBV-positive epithelial cells, but not in the lymphoma. It is also not restricted during the lytic cycle, but also during the latency period [78].

LMP1 induces expression of intracellular adhesion molecules 1 (ICAM-1), CD40, and pro-inflammatory cytokines, such as interleukin-6 (IL-6) and IL-8 [51,79]. Overexpression of LMP1 has been shown to worsen survival ratio and increase invasiveness in an immortalized NPE cell line [80]. LMP1 reactivates EBV and regulates the EBV gene expression in differentiating epithelial cells [81,82]. These suggest that tight regulation of LMP1 may reflect the co-evolution of the oncogenesis, immune evasion, and viral pathogenesis in the host cells. LMP1 is also known to induce cell cycle disruption and genomic instability in NPC cells [83,84].

In addition, non-coding RNAs (ncRNAs) are parts of the EBV transcriptome, which are transcribed during various life cycle stages. EBV ncRNAs consist of EBERs, small nucleolar RNAs (snoRNA1), and microRNAs (*Bam*H I fragment H rightward open reading frame miR-(BHRF)s and miR-BARTs) [85]. These ncRNAs differentially regulate host targets. While EBER and snoRNA form a secondary structure which facilitates the interaction to signaling proteins directly [86], BHRFs and BARTs interact with RNA-induced silencing complex (RISC) as a guide for complementary mRNA target that leads to its degradation [87]. During the last decade, a large number of EBV ncRNAs have been reported and investigated for their roles in NPC [88].

The host factors and genetic modification are important for maintaining EBV activity. It has been shown that EBV is unable to transform the primary and immortalized NPE cells. Rather, immortalized NPE cells infected with EBV become cell senescence and stop cell division [89]. However, cells lacking p16, a negative regulator of cyclin D1, or overexpressing cyclin D1, can grow while maintaining EBV infection [89]. It has been reported that premalignant nasopharyngeal epithelium overexpressed cyclin D1, which enables persistent infection of EBV [89,90]. The overexpression of polycomb complex protein, B lymphoma Mo-MLV insertion region 1 homolog (Bmi-1), is able to immortalize primary epithelial cells, which allows the EBV to establish latency [91,92]. These indicate the interplay of host factors in regulating the EBV proteins in the infected epithelial cells.

### 4.3. EBV Lytic Infection in NPC

The EBV life cycle includes both the lytic and latent infection. Lytic infection is frequently associated with apoptosis [93]. First, the expression of immediate-early (IE) genes such as BZLF1 and BRLF1 are induced by cellular stress. In turn, the expression of early genes including viral protein kinase (BGLF4), viral DNase (BGLF5), viral DNA polymerase (BALF5), EA, BALF3, BARF1, and BHRF1 are induced by BZLF1 and BRLF1. Finally, late genes such as BCRF1 and VCA are highly expressed. These lytic proteins are involved in the amplification and replication of the viral genome [93]. Interestingly, small populations of cells showing lytic infection are commonly found in EBV-infected patient-derived xenograft cell lines [67,94]. Consistently, high-risk NPC patients exhibited elevated antibody titers against EBV lytic proteins, such as BGLF5, BALF5, EA, and VCA [95,96].

BZLF1 induces the expression of inflammatory cytokines such as IL-8 [97]. Though increased inflammation can be observed in NPC, cells showing lytic infection of EBV can evade CD8+ T cell recognition [98]. Moreover, EBV lytic proteins including BGLF4, BGLF5, and BALF3, could induce chromosomal aberration, DNA damage, and genomic instability [99,100,101]. Other lytic proteins, such as BARF1, BHRF1, and BCRF1, can also induce an anti-apoptotic phenotype [102]. These suggest that EBV lytic proteins enhance the tumorigenicity of NPC cells.

## 5. EBV-Mediated Signaling

**Figure 3:**
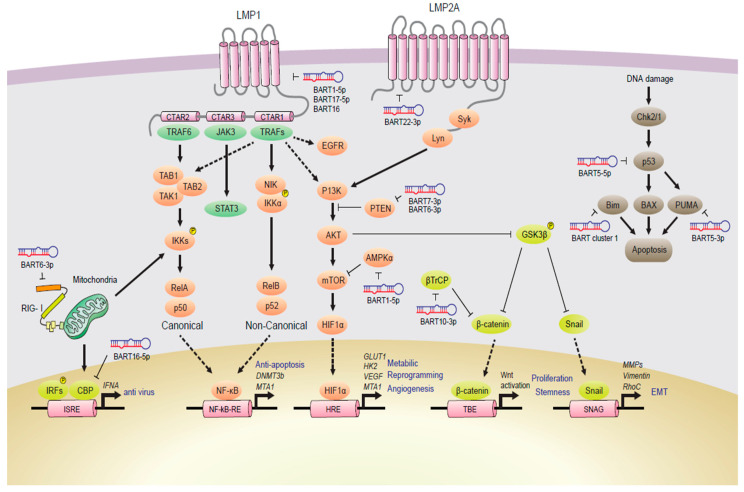
EBV-mediated signaling in NPC. An overview of signaling pathways in the NPC mediated by EBV infection. The EBV-encoded proteins including LMP1, LMP2A, and EBNA1 cause dysregulation in intracellular signaling pathways in NPC cells. This figure represents the signaling pathways, such as WNT/β-catenin, JAK/STAT, PI3K/Akt/mTOR, EGFR, and MAPK, and NF-κB, of which details are described in text. EBV ncRNA can also regulate these signals and enhances tumorigenesis and progression of NPC. The headed arrows show up-regulation and blunt-ended arrows show downregulation.

Dysregulation of intracellular signaling mediated by EBV during NPC carcinogenesis has been evident and described. The EBV-mediated signaling pathways in NPC are depicted in Figure 3.

### 5.1. Wnt/β-Catenin

Wnt signaling pathway is a result of Wnt ligand interacting with a corresponding membrane receptor, thereby causing a nuclear translocation of β-catenin and triggering gene expression. The canonical Wnt pathway plays a role in the embryonic process and contributes to carcinogenesis and cancer progression [103,104,105]. β-catenin acts as a pivotal protein and its overexpression has been observed in cancer stem cells (CSCs) and many tumors including NPC [106]. Wnt signaling pathway is dysregulated by EBV during NPC progression. The expression of LMP1 is not correlated with the activation of β-catenin in the canonical Wnt pathway [107]. On the other hand, LMP2A induces MTA1, which stimulates the downstream Wnt cascade, such as phosphorylation of glycogen synthase kinase 3-beta (GSK3β) and nuclear translocation of β-catenin, thereby LMP2A enhances EMT process and tumor invasion [76]. Moreover, LMP2A activates the non-canonical Wnt pathway, inducing growth, and progression of NPC [108].

### 5.2. JAK/STAT

Janus kinases (JAK)-signal transducer and activator of transcription (STAT) signaling is composed of JAK, STAT, and cellular receptors. When cytokines bind to cellular receptors, JAK becomes phosphorylated and then phosphorylates STAT. Receptor-associated STAT is phosphorylated by JAK. Phospho-STAT is then translocated into the nucleus and regulates immune response. STAT protein members such as STAT1, STAT3, and STAT5 are upregulated in NPC tissues [109]. In addition, JAK1, a member of JAK family, promotes Qp activity of EBNA-1 and promoter of LMP1 by binding of activated STAT proteins in vitro [109,110]. JAK-STAT signaling pathway is triggered by the LMP1 through its carboxyl-terminal activation domain 3 (LMP1-CTAR3), which then activates JAK3 and promotes its phosphorylation [111], resulting in nuclear translocation [111,112,113]. STAT3 also binds to the cyclin D1 promoter, which enhances its transcription [114]. In addition, STAT3 promotes angiogenesis as it is a transcriptional-activator of the vascular endothelial growth factor (*VEGF*) gene [115]. Overexpression of STAT3 has been shown to link to VEGF and exhibits a negative correlation with the survival rate in NPC patients [116].

### 5.3. PI3K/Akt/mTOR

Phosphoinositide 3-kinase/protein kinase B (PI3K)/Akt cascade plays a crucial role in the NPC carcinogenesis via activation of EBV oncoproteins such as LMP1 and LMP2A [117]. LMP1 stimulates the PI3K/Akt pathway, which contributes to the induction and maintenance of CD44-positive CSC properties. PI3K/Akt and LMP1 create a positive feedback loop to regulate LMP1-induced CSC in NPC cell population [118,119]. A PI3K inhibitor, LY294002, has been shown to inhibit NPC cell proliferation and induces apoptosis, revealing PI3K as a potential target for therapeutics [120]. The mammalian target of rapamycin (mTOR) is a downstream player of the PI3K/Akt pathway. It phosphorylates and activates its effectors such as ribosomal protein S6 kinases (P70S6K) and eukaryotic initiation factor 4E binding protein (4EBP1). It has been shown that LMP1, p-P70S6K, and p-4EBP1 are up-regulated in NPC clinical samples [121], but are negatively correlated with NPC patient outcome [122]. EMT can be initiated through the epithelial cell adhesion molecule (EpCAM), which is highly upregulated in both RNA and protein levels in NPC. EpCAM participates in CSC phenotypes in vitro and cellular metastasis by enhancing mTOR signalings, such as Akt, mTOR, P70S6K, and 4EBP1 [123], and is also related to survival rate in NPC patients. Furthermore, a tumor suppressor protein, WW domain-containing oxidoreductase (WWOX), which is regulated by LMP1, has been shown to be correlated with the TNM stages of NPC [124]. LMP2A also enhances the migrative and invasive properties and the expression of stem-like cell markers in NPC through AKT pathway activation [125].

### 5.4. EGFR and MAPK

The epidermal growth factor receptors (EGFRs) are tyrosine kinase receptors, whose dysregulation has been reported in various epithelial tumors. Co-expression of LMP1 and EGFR is found in more than 60% of NPC clinical samples and is correlated with poor prognosis [126]. The LMP1-CTAR1 activates NF-κB family members, p50/p50 and Bcl-3 complexes, and upregulates EGFR [127]. LMP1 also enhances phosphorylation and nuclear translocation of EGFR [128], which is crucial in cell proliferation and differentiation associated with mitogen-activated protein kinases (MAPK). MAPK pathway, which is known as the Ras-Raf-MEK-extracellular signal-regulated kinase (ERK) pathway, transmits the signal from a receptor on cell surface into the nucleus via phosphorylation activity of numerous kinase proteins. MAPK pathway is dysregulated in LMP1-overexpressing NPC cells [121]. LMP1-CTAR1 also activates EGFR and ERK through protein kinase C delta (PKC delta) [113], which promotes NPC cell motility and invasiveness through activation of the ERK-MAPK pathway [129]. In addition, LMP1-CTAR1 induces the transcription of hypoxia-inducible factor 1-α (HIF-1α) via recruitment of ERK1/2 and NF-κB [130]. LMP1-positive extracellular vesicle from EBV-positive NPC enhances recipient NPC cell proliferation, invasion, and radioresistance through P38 MAPK pathway [125]. Furthermore, LMP2A upregulates EGFR, and intracellular Ca^2+^, which promotes Ca^2+^-dependent protease and calpain that cleave integrin β4 (ITGβ4) from the basal layer to peripheral membrane structures, leading to the motility of NPC cells [131].

### 5.5. NF-κB

NF-κB functions as a transcription factor forming several dimeric combinations, including NF-κB1 (p50/p105), NF-κB2 (p52/p100), c-Rel, RelA (p65), and RelB. The NF-κB is regulated by IκBs, which are phosphorylated by IκB kinases (IKKs). The phosphorylation and subsequent degradation of IκBs enable NF-κB to translocate to the nucleus and regulates transcription of various genes involved in the inflammation, immunity, stress responses [132]. Somatic mutation in genes for NF-κB regulators has been observed in NPC cells, with dominant alteration in NF-κB inhibitor alpha (*NFKBIA*), CYLD lysine 63 deubiquitinase (*CYLD*), and tumor necrosis factor receptor-associated factor 3 (*TRAF3*) [27,133,134]. *NFKBIA* encodes IκBα. CYLD negatively regulates NF-κB by deubiquitinating target proteins, such as IKKγ (NEMO), TRAF2, and TRAF6 [134]. CYLD overexpression strongly suppressed the growth, proliferation, metastasis, and migration of NPC cells [135]. It has been reported that dysregulation of NF-κB by somatic mutations and LMP1 overexpression are mutually exclusive in NPC development [27].

LMP1 activates NF-κB pathway through recruitment and activation of TRAFs, TRADD, EGFR, and others [136,137]. LMP1 increases phosphorylation of IκB then increases nuclear translocation of NF-κB to activate target genes [138]. The effect of EBV infection in NPC cells appears to be leaning towards regulation over NF-κB activity rather than the expression of NF-κB signaling proteins. Interestingly, the IκBα phosphorylation by IKKβ is induced by LMP1 activation via mTORC1 pathway, with further modulation on aerobic glycolysis through Glut-1 transcription [139]. Constitutive activation of NF-κB is a key step in NPC development.

LMP1 promoter is also activated by NF-κB through the p50-p50 homodimer and the p65-p50 heterodimer [140]. The CTAR1 of LMP1 can activate p50-p50, p50-p52, and p52-p65 dimers through TRAF-1, 2, and 3, whereas the CTAR2 activates p52-p65 through tumor necrosis factor receptor-associated death domain protein (TRADD) and TRAF2 [141,142]. Moreover, the p52-RelB complex requires the NF-κB-inducing kinase (NIK) and IKKα interaction and translocate to the nucleus [143].

The EBV-encoded BARTs miRNAs are regulated by the interaction of p50 subunit to NF-κB site at the BART promoter. BARTs miRNA expression upregulates LMP1 level in EBV-infected NPC cells [144]. Considering the dynamic of BART and LMP1 via NF-κB, the establishment of latent EBV infection forms an oncogenic stimulatory loop in NPC cells. In addition, LMP1 inhibits phosphatase and TEN-sin homolog (PTEN) expression, because LMP1 upregulates DNA methyltransferase 3b (DNMT3b) via activation of p65 subunit, which hypermethylated the PTEN promoter [145]. The p65 subunit can also bind to the Qp EBNA1 promoter and upregulate EBNA1 expression [146].

## 6. Cancer Hallmarks of EBV-Associated Malignancies

**Figure 4:**
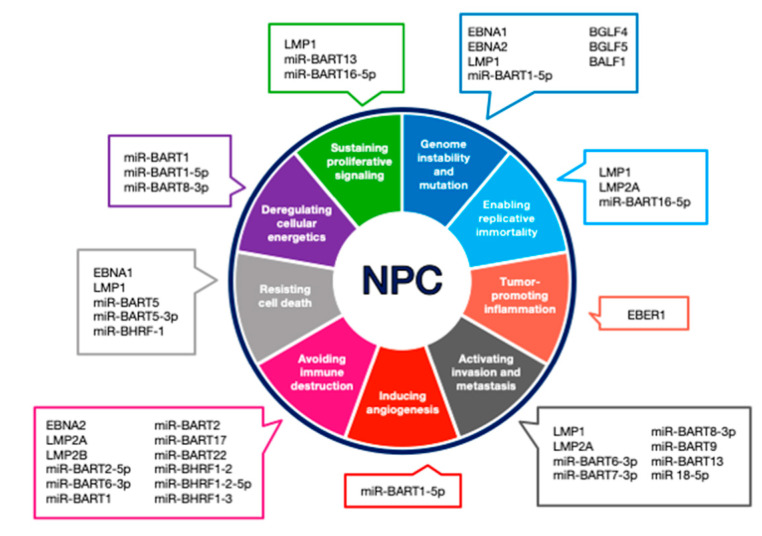
Malignancies associated with EBV. Summary of EBV major proteins, antigens, and microRNAs involved in establishment of NPC adapted from hallmarks of cancer established by Hanahan and Weinberg [147].

The core features of cancer have been well explored in tumor biology, and it has been further developed and revisited throughout the years. The features correspond to the hallmarks elucidated by Hanahan and Weinberg [147], described below and in Figure 4 are the hallmarks associated with EBV in NPC development.

### 6.1. Immune Evasion

Immune evasion is a survival key for cancer cells. EBV sets a limited viral protein to be expressed during latency and to shed, establishing balance hiding from the host immune signaling pathway. LMP1 represses the expression of TLR9 whose ligand is viral unmethylated CpG DNA through activation of NF-κB [148]. LMP1 also promotes SUMOylation of interferon regulatory factor 7 (IRF7), which is important for the induction of type I interferon (IFN) [149]. On the other hand, LMP2A and 2B suppress IFN signaling by promoting the degradation of IFNα and IFNγ receptors [150]. Furthermore, many EBV lytic proteins are detected in NPC cells or patients, which are involved in immune evasion.

Likewise, EBV ncRNAs have been shown to allow NPC cells to avoid immune cell surveillance and escape the immune system [151,152]. Pattern recognition receptors play an important role in the innate immune response by detecting viral pathogens. During EBV infection, EBV miR-BART6-3p targets the binding sites in the 3′ untranslated region (UTR) of retinoic acid-inducible gene I (RIG-I) mRNA, resulting in a reduction of type I IFNs response [153]. Type I IFN signaling can also be inhibited at the transcription level via EBV miR-BART16-5p, which directly targets the 3′UTR of CREB-binding protein mRNA, a co-activator of IFNs [154]. Both EBV miR-BART6-3p and EBV miR-BART16-5p halt anti-proliferative properties of type I IFN, thereby enhance the tumorigenicity of NPC cells.

The cytokine network is a regulator of innate and adaptive immune systems [155]. Thus, disruption in cytokine signaling provokes NPC progression. EBV miR-BHRF1-2-5p targets the 3′UTR of IL-1 receptor mRNA, thereby reducing an early activation of the immune system from IL-1 signaling. Because EBV miR-BART1, BART2, and BART22 reduce IL-12 production, NK cells are inactivated and the transformation of immature CD4^+^ T cells to mature helper T cells is suspended, and activation of CD4^+^ effector T cells is reduced [156]. In addition, other proteins in cytokine production such as Cathepsin B, a protease in the antigen-presenting process, are targeted by EBV miR-BHRF1-2. An antigen peptide transporter 2 (TAP2) can also be disrupted by EBV miR-BHRF1-3 and BART17 [157]. EBV-infected cells evade the immune cells because EBV miR-BART2-5p directly targets the 3′UTR of MHC class I polypeptide-related sequence B (MICB) mRNA, a key ligand for NKG2D receptor in stress-induced response. Consequently, cancer cells can escape from NK cells by the loss of MICB [158]. Interestingly, RIG-I receptor can recognize the secondary structure of EBER1, which relays the signal via RIG-I/IRF-3 axis, resulting in the upregulation of IL-10 that promotes cancer cell growth [159]. To summarize the above, EBV infection can modify antigen recognition pattern and cytokine production that promotes NPC carcinogenesis through compromising the immune system.

Nevertheless, the strategies on how the virus initially escapes the immune response remain poorly understood.

### 6.2. Metabolic Reprogramming

The links between EBV and metabolic reprogramming have been elucidated. EBV ncRNAs manipulate metabolic pathways via suppression of metabolic genes or inhibition of LMP1 [160]. Overexpression of EBV miR-BART1 reduces the expression of glucose-6-phosphate dehydrogenase (*G6PD*)*,* spermidine/spermine N1-acetyltransferase 1 (*SAT1*)*,* arginosuccinate synthetase gene (*ASS1*), and other metabolic genes [161]. EBV miR-BART1-5p also directly targets the 3′UTR of AMP-activated protein kinase alpha 1 gene (*AMPKa1*) mRNA, which consequently activates AMPK/mTOR/HIF1 pathway and its downstream metabolic genes including glucose transporter 1 (*GLUT1*) and hexokinase 2 (*HK2*) gene. Because VEGF is also expressed via AMPK/mTOR/HIF1 axis, EBV miR-BART1-5p may promote both metabolic reprogramming and angiogenesis in NPC [162]. Therefore, suppression of EBV miR-BART1 will reduce the metabolic and vascular formation phenotypes of the NPC cells. Recently, EBV-miR-BART8-3p also promotes radioresistance by modulating the activity of the ATM/ATR signaling pathway [163].

EBV miR-BART1-5p, BART16, BART17-5p, and BART cluster I can indirectly modulate glycolysis by targeting LMP1 expression. GLUT1 is an example [160]. CTAR1 of LMP1 activates mTORC1 through AKT/ERK/IKKα. On the other hand, CTAR2 regulates mTROC1 via IKKβ. The mTORC1 then triggers GLUT1 expression through NF-κB signaling [139]. LMP1 also upregulates HK2 via stabilization of the HK2 transcription factor, c-MYC. c-MYC can be phosphorylated by GSK3β and is usually subject to degradation by proteasome. However, LMP1 inactivates GSK3β through PI3K/Akt signaling and stabilizes c-MYC. Consequently, overexpression of HK2 promotes glycolysis and mitochondria-dependent apoptosis [164]. In addition to glycolysis, aberrant regulation of lipid metabolism is also mediated by EBER. Overexpression of EBER leads to the upregulation of low-density lipoprotein receptor (LDLR) and fatty acid synthase (FASN). Moreover, EBER overexpression induces a lipoprotein-dependent proliferation phenotype in NPC cells [165].

### 6.3. Apoptosis

Apoptosis is a programmed cell death, which is a major event in the cancer hallmarks [166]. The apoptotic pathway can be triggered via many signaling axes, such as an extrinsic pathway with death receptors through the tumor necrosis factor (TNF) or mitochondria-mediated cytochrome C pathway. Many players in these signaling pathways can be targeted by EBV miRNAs, leading to modulation of apoptosis during EBV infection [71].

The p53 is a transcription factor with pro-apoptotic properties. When DNA damages occur, p53 is phosphorylated via ATM/chk1/2 axis. Phosphorylated p53 then promotes cell cycle arrest to repair the damaged lesion. Therefore, the malfunction of p53 disrupts cell cycle arrest and allows cells to grow uncontrollably [167]. Low expression of PUMA is found in 60% of human NPC tissues. It has been shown that EBV miR-BART5 targets the 3′UTR of *PUMA* mRNA, resulting in the loss of mitochondrial apoptotic signaling in NPC cells [168]. EBV miR-BART5-3p and miR-BHRF-1 have been shown to inhibit p53 directly by targeting the 3′UTR of *TP53* mRNA. As a result, EBV miR-BART5 enhances cell cycle progression and inhibits apoptosis [169,170]. Furthermore, Bcl-2 interacting mediator of cell death (BIM), a pro-apoptotic protein in the BAX induced mitochondrial apoptosis, is also downregulated by EBV miR-BARTs in both cluster I (miR-BART1 and BART3) and cluster II (miR-BART9, BART11, and BART12) [171]. In addition, EBNA1 interacts with USP7, the regulator of p53, and depletes p53 through proteasome degradation. EBNA1 also blocks caspase activation through Survivin [172,173]. LMP1 triggers resistance in NPC cells through PI3K/Akt/FOXO3a pathway by altering human miR-21 expression [174].

### 6.4. Metastasis

Cancer metastasis is a prominent feature in many NPC cases and is a major cause of poor survival in patients [175,176,177]. EBV DNA load has been used to estimate the prognosis of patients undergoing therapy and to diagnose the risk of metastasis [178]. EMT is a phenotypic change from epithelial cells to mesenchymal cells by reorganizing cell polarity, cell-cell interaction, and cell-extracellular matrix adhesion [161]. LMP2A promotes malignancy of NPC cells through the induction of matrix metalloproteinase 9 (MMP-9) [179]. Both LMP1 and LMP2A reduce the type IV collagen at the basement membrane and induce stemness-like cell growth by expressing BMI1, SOX2, and stem cell-related surface markers (CD44v6 and CD133) through the hedgehog signaling pathway. Thus, EBV oncoproteins are crucial for the maintenance and development of stemness in NPC [180]. Several EBV miRNAs have been reported to modulate the EMT process and enhance the metastatic process in NPC cells [85]. High expression of EBV miR-BART7-3p is correlated with lymph node metastasis and the clinical stage of NPC. EBV miR-BART7-3p, miR-BART9, and miR-18-5p downregulate PTEN by directly targeting 3′UTR of PTEN mRNA, which leads to translocation of Snail and β-catenin to the nucleus. PTEN knockout mimics the phenotypes of EBV miR-BART7-3p in NPC cells, while PTEN complementation reverses such phenotypes [181,182]. In addition, EBV miR-BART6-3p contributes to EMT progression by blocking cell migration via the downregulation of lncRNA LOC553103 [183]. EBV-miR-BART8-3p induces EMT by activating the NF-κB and ERK1/2 pathway [184]. On the other hand, EBV miR-BART10-3p promotes cell invasion through modulation of the stabilizer complex beta-transducin repeat containing E3 ubiquitin protein ligase (βTrCP), ZEB1, N-cadherin, Vimentin, and Slug transcription factors [185]. Promoting metastasis is not the sole function of EBV miRNAs, EBV-miR-BART13 promotes cell growth by targeting the NK1RAS2/NF-κB pathway [186].

Alteration of cell adhesion molecules is also involved in NPC cell invasion [187]. E-cadherin is a transmembrane protein that connects epithelial cells via adherent junction and loss of E-cadherin transforms pre-cancerous cells to invasive and metastatic cells [188]. EBV miR-BART9 binds to the 3′UTR of E-cadherin mRNA, thereby reducing E-cadherin production. Taken together, E-cadherin expression and EBV miR-BART9 could be effective biomarkers that predict the aggressiveness of NPC [189].

### 6.5. Sustaining Proliferative Signal

The human telomerase reverse transcriptase (hTERT) is a catalytic subunit of telomerase that maintains and extends telomeres in the eukaryotic chromosome, thus induces replicative senescence of cells and immortalizes cancer cells [190]. LMP1 mainly sustains proliferative signals via NF-κB pathway. LMP1 promotes nucleus translocation of NF-κB p65 then nuclear accumulation of hTERT, which upregulates telomerase activity and cell proliferation [191,192,193]. An increase in expression and phosphorylation of hTERT has been reported in LMP1-positive NPC cells [132,194], with the involvement of c-Myc [195]. Similarly, CTAR1 and CTAR2 domains of LMP1 also activate telomerase. NF-κB activation plays an important role for immortalization of NPE cells [196] with involvement of upstream EGFR/MEK/ERK/IKK/mTORC1 activation [197]. Both LMP1 and hTERT expression are important in immortalization of NPE cells [198]. It has been revealed that LMP1-NF-κB dynamic inhibits the expression of PINX1, an inhibitor to telomerase [199]. In addition to NF-κB pathway, telomerase activity is also induced by LMP1 through p16INK4A/Rb/E2F1 and JNK signaling pathways [200].

## 7. Conclusions

This review covers a recent understanding of the pathogenic role of EBV infection in NPC carcinogenesis. It is evident that EBV oncoproteins initiate the tumorigenesis via modulation of multiple signaling pathways including Wnt/β-catenin, JAK/STAT, PI3k/Akt/mTOR, EGFR/MAPK, and NF-κB pathways. In addition, the regulation of certain signaling pathways has been demonstrated by EBV ncRNA as summarized in Figure 4. Further investigations are required to identify the exact signaling pathways of EBV oncoproteins/ncRNA interactions and their roles in NPC initiation and progression. These molecules could provide new targets for the development of NPC therapeutics.

## Figures and Tables

**Table 1:** EBV strains from NPC tissue.

EBV Genomes	Sources	GenBank Accession Number	LMP1 Strains	References
GD1	Saliva of NPC patients	AY961628	China 1	[44]
GD2	Biopsy of NPC patients	HQ020558	China 1	[45]
M81	LCL isolated from NPC patients	KF373730	China 1	[46]
HKNPC1	NPC patient	JQ009376	China 1	[47]
HNKPC2	NPC patient	MH590513	China 1	[47]
HNKPC3	NPC patient	MH590514	China 1	[47]
HNKPC4	NPC patient	MH590515	China 1	[47]
HNKPC5	NPC patient	MH590516	China 1	[47]
HNKPC6	NPC patient	MH590517	China 1	[47]
HNKPC7	NPC patient	MH590518	China 1	[47]
HNKPC8	NPC patient	MH590519	China 1	[47]
HNKPC9	NPC patient	MH590520	China 1	[47]
C666-1	NPC cell lines	KC617875	China 1	[48]
M-ABA	LCL isolated from NPC patients	LN827527	B95.8	[49]
